# Preeclampsia, the placenta, ethnicity, and social determinants of health

**DOI:** 10.1111/aogs.14939

**Published:** 2024-08-12

**Authors:** Meryam Sugulle

**Affiliations:** ^1^ Faculty of Medicine University of Oslo Oslo Norway; ^2^ Division of Obstetrics and Gynecology Oslo University Hospital Oslo Norway

Preeclampsia, a placenta‐mediated pregnancy complication that globally affects an estimated 2%–5% of women,^1^ may severely impact both maternal and fetal–neonatal morbidity and mortality in both the short and long term. Potentially lethal complications for the woman include eclampsia and cerebral hemorrhage as well as increased risk for future diabetes mellitus and cardiovascular disease, alongside preterm birth, fetal growth restriction, and stillbirth for the baby, all further magnified in early‐onset disease before 34 gestational weeks.^1^ Prevention of preterm preeclampsia in high‐risk women by means of aspirin prophylaxis has proven to be effective.^2^ In light of the potentially severe health consequences of preeclampsia, identification of women at increased risk is endorsed by the International Federation of Gynecology and Obstetrics, and first‐trimester screening is currently established in many countries.^1^

Still, the question remains whether currently available screening algorithms are globally valid and whether they can be further improved?

Risk factors inform risk prediction models in the sense that these models usually are constructed by applying several risk factors based on patient characteristics associated with the health outcome of interest. Screening algorithms for preeclampsia, such as the Fetal Medicine Foundation (FMF) combined algorithm,^3^ include the “Racial origin” of the pregnant woman, categorized as “White,” “Black,” “South Asian,” “East Asian,” and “Mixed.”

Categorization of “race” is commonly done by self‐identification, for example based on skin color (“Black” vs “White”) and so being, a “social construct.”^4^, ^5^ Categorization of ethnicity refers partly to geographical denominations/country of birth of oneself or one's mother/father, relying on the assumption of an implicitly shared cultural background.^4^ The terms “race” and “ethnicity” are often being used interchangeably despite their differing definitions.^4^

Studies on the association between “race” and ethnicity and preeclampsia risk are contradictory. In the UK, “Black” women, predominantly with parents from the Caribbean, Nigeria, or Ghana, and South Asian women have been identified as at‐risk populations for developing preeclampsia, compared with “White” women.^6^ In contrast, in Norway, where categorization by country of birth is applied, a lower risk of preeclampsia/eclampsia and gestational hypertension in foreign‐born women (including countries where people would self‐identify as “Black” or “South Asian”) was found.^7^ In Norway, if “race” were to be applied, “Black” women would predominantly have Somalia as own or parental country of origin, in contrast to the above study from the UK.^6^, ^8^ This is one example illustrating that comparisons between studies from different regions with regard to apparently same categories of “race,” cannot necessarily be made. Furthermore, a recent Swedish Register‐based study comparing the risk of hypertensive diseases of pregnancy (HDP) among first‐ and second‐generation immigrant women compared with Swedish women showed an overall lower risk of HDP among the immigrant women.^9^

Limitations related to the analysis and comparison of data regarding ethnicity lie in the large variety of ethnic groups: firstly, not all ethnic groups may be accurately defined and secondly, the limited number of study participants for some ethnicities leads to the merging of quite diverse ethnic groups into more or less randomly generated larger “ethnic collections.” As an example, several thousand ethnicities exist in Africa alone.

The FMF screening algorithm has been tested in various geographical settings spanning different ethnicities, and variations in gestational medians for placental growth factor (PlGF) concentration in maternal circulation included in the risk assessment algorithm have been noted.^1^, ^10^, ^11^ Wright et al. report higher maternal PlGF and lower sFlt‐1/PlGF ratio in “Black” compared with “White” women, and point out that fixed cut‐offs for angiogenic biomarkers may lead to underestimation of increased preeclampsia risk in “Black” women.^11^ The authors express the need for further studies of angiogenic factors in pregnant women classified as “Black” from a variety of regions in order to test the external validity of their finding.^11^ One proposed hypothesis for the “racial” differences in maternal circulating angiogenic markers is a higher placental production,^12^ implying the suggestion of a genetic basis. Maternal “race—not” “placental race or ethnicity” informs risk assessment and screening algorithms. To illustrate: What “race” or ethnicity do a fetus and the placenta have when the mother is for example of North European descent, “White,” and the father of East African heritage, “Black”? Depending on the categorization method, the answer will either be North European or “Black” or “Mixed race.” A US study of genetic variation in different angiogenic pathway genes and preeclampsia in “Black” and “White” women revealed allelic variants in both groups associated with preeclampsia; however, these variants were not associated with dysregulated maternal circulating angiogenic biomarker levels and presence of disease within the groups.^13^

A recently published guidance by Feero et al. proposed to abstain from the use of self‐identified race and ethnicity as analytic variables based on the notion that individual genetic variation is insufficiently captured by existing population descriptors.^5^

It is widely acknowledged that social determinants of health are mediators of health outcomes in general, and this also applies to adverse pregnancy outcomes.

Minopoli et al. recently showed in a study of more than 13 000 singleton pregnant women attending routine first‐trimester FMF algorithm screening for preeclampsia, that despite “White” women having a lower prevalence of composite adverse pregnancy outcomes, half of all these outcomes affected women who were classified as “White” and were associated with socioeconomic deprivation.^14^ The authors concluded that both ethnicity and socioeconomic deprivation influenced the prevalence of placenta‐mediated adverse pregnancy outcomes, and that there exists a strong interaction between those two risk factors.^14^ Accordingly, the authors proposed the inclusion of socioeconomic deprivation in risk assessment for pregnancy complications such as preeclampsia.^14^ Minopoli et al. pointed out that there is a danger of giving the supposedly “lower‐risk” group of “White” women based on “ethnic risk evaluation” less good health care when the context of social determinants is not taken into account.^14^ In accordance with the above, Wändall et al. showed that Swedish‐born women living in middle‐ and high‐deprivation areas with two Swedish‐born parents were at increased risk of HDP.^9^

Arechvo et al. demonstrated recently that adding indices of multiple deprivation (IMD) to the FMF history‐only competing risks model preeclampsia screening algorithm did not improve the prediction of preeclampsia for women living in England.^15^ The authors confirm the association between social deprivation and higher incidence of preeclampsia, and acknowledge both the limitations of the categories of “race” as well as of the IMD as a measure of relative deprivation.^15^

Further disentangling the effects of socioeconomic factors and ethnicity and their respective predictive value in screening algorithms should be the subject of future studies.

Study designs should account for maternal or parental country of birth, since one distinct category of “race” in one region of the world might include a completely different population as compared to another region, as argued above. Also, information on country of birth should rather be considered a risk marker warranting further investigation into socioeconomic status and access to and usage of health care services instead of assuming biological causality. Socioeconomic deprivation might impact pregnancy outcomes differently depending on the region studied and their respective health care systems (US vs European countries). Further research into associations between maternal and/or parental country of birth and maternal circulating angiogenic biomarker gestational medians could clarify whether maternal ethnicity is relevant for the evaluation of placenta‐associated biomarkers in case of fetuses of “mixed” origin. Our foremost societal aim should be to facilitate good health for as many people as possible. Therefore, we must take care to advance health research by discerning the factual determinants of health—so as to better see the whole picture.
